# Drug-induced liver injury secondary to rituximab use

**DOI:** 10.1093/omcr/omaf003

**Published:** 2025-07-14

**Authors:** Samantha Melanie Armendariz-Pineda, Andres Manuel Vargas-Beltran, Maria Juliana Corredor-Nassar, Armando Gamboa-Domínguez, David Arturo Santos-Reyes, Jacqueline Cordova-Gallardo

**Affiliations:** Faculty of Medicine, National Autonomous University of Mexico, 411A Escolar Street, Copilco Universidad, Coyoacan, 04360 Mexico City, Mexico; Department of Hepatology, Internal Medicine Service, Hospital General Dr. Manuel Gea Gonzalez, 4800 Calzada de Tlalpan, Belisario Domínguez Section 16, Tlalpan, 14080 Mexico City, Mexico; Department of Hepatology, Internal Medicine Service, Hospital General Dr. Manuel Gea Gonzalez, 4800 Calzada de Tlalpan, Belisario Domínguez Section 16, Tlalpan, 14080 Mexico City, Mexico; Faculty of Medicine, Meritorious Autonomous University of Puebla, 2702 13 Sur Street, Los Volcanes, 72420 Heroica Puebla de Zaragoza, Mexico; Internal Medicine Service, Hospital General Dr. Manuel Gea Gonzalez, 4800 Calzada de Tlalpan, Belisario Dominguez Section 16, Tlalpan, 14080 Mexico City, Mexico; Department of Pathology, Instituto Nacional de Ciencias Médicas y Nutrición Salvador Zubirán, 15 Vasco de Quiroga, Belisario Dominguez Section 16, Tlalpan, 14080 Mexico City, Mexico; Department of Pathology, Instituto Nacional de Ciencias Médicas y Nutrición Salvador Zubirán, 15 Vasco de Quiroga, Belisario Dominguez Section 16, Tlalpan, 14080 Mexico City, Mexico; Faculty of Medicine, National Autonomous University of Mexico, 411A Escolar Street, Copilco Universidad, Coyoacan, 04360 Mexico City, Mexico; Department of Hepatology, Internal Medicine Service, Hospital General Dr. Manuel Gea Gonzalez, 4800 Calzada de Tlalpan, Belisario Domínguez Section 16, Tlalpan, 14080 Mexico City, Mexico

**Keywords:** drug-induced liver injury, rituximab, ursodeoxycholic acid, prevention

## Abstract

Abbreviations: DILI: drug-induced liver injury, HBV: Hepatitis B virus, LFT: liver function test, RRMS: Relapsing–remitting multiple sclerosis, UDCA: ursodeoxycholic acid. Rituximab is a human chimeric monoclonal antibody that targets CD20 on B cells. The incidence of rituximab-associated drug-induced liver injury (DILI) is 19 cases per 100 000 people annually. This condition is characterized by a rapid increase in aminotransferase levels and hepatocellular injury, primarily due to reactivation of the hepatitis B virus (HBV). Nevertheless, there have been rare cases of DILI occurring in the absence of HBV reactivation. This case presents a 25-year-old male with relapsing–remitting multiple sclerosis (RRMS) that was treated with rituximab. After treatment, the patient demonstrated a hepatocellular damage pattern and biopsy confirmed the diagnosis. Following the administration of ursodeoxycholic acid (UDCA), the patient’s liver function tests (LFTs) returned to normal levels, demonstrating that DILI from rituximab can occur after any infusion, regardless of therapy duration or dosage.

## Introduction

Rituximab is a human chimeric monoclonal antibody directed against CD20, a glycoprotein present on the surface of B cells, that is used in the treatment of B cell lymphomas and various autoimmune diseases [1]. Toxic effects related to this drug are usually mild to moderate and generally occur with the first infusion dose [2]. Drug-induced liver injury (DILI) associated with rituximab has an annual incidence of 19 cases per 100 000 inhabitants per year. It is associated with reactivation of HBV and represents a higher risk of occurrence in women (56%–59%) than in men (41%–44%) [3, 4]. This increased risk of DILI in women has been thought to be due to several factors, including hormonal influences, differences in drug pharmacokinetics and pharmacodynamics, variations in the immune system responses to specific drugs, the presence of reactive metabolites or drug-protein complexes, and interactions with immunomodulatory agents or signaling pathways [5]. Clinically, DILI is characterized by a rapid and abrupt onset of elevated aminotransferase levels, 15 to 20 times the upper normal limit, and hepatocellular injury secondary to HBV reactivation [2]. It has been the most frequently documented association in the literature. However, there have been very few reported cases of DILI occurring independently of HBV reactivation.

**Figure 1:**
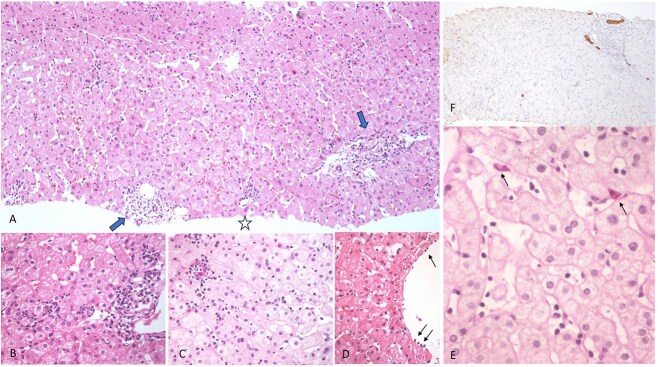
Acute liver damage without cholestatic changes. A. Panoramic view with inflammatory infiltrate in portal triads with edema (arrows). Endotheliitis in central vein (star) and sinusoidal inflammation with discrete congestion (HE, 100X). Lymphocytes surround hepatocytes with degenerative changes in B and C (HE and diastase PAS, both X400). Lymphocytes attached to endothelial cells in a medium size central vein (HE X400, thin arrow). D-PAS showing pigmented Kupffer cells in E (X400). Cytokeratin 7 decorates ductules and a cholangiole without reaction in hepatocytes (X40).

**Table 1:** Liver function tests evolution

**Liver function Tests**											
							**UDCA** **START**	**UDCA**			
	**June 7, 2021** **1st infusion**	**July 18, 2021** **2nd infusion**	**January 12, 2022** **3rd infusion**	**July 17, 2022** **4th infusion**	**January 16, 2023** **Hepatology service**	**January 24, 2023** **Liver Biopsy**	**February 10, 2023** **DILI RTX**	**March 21, 2023** **5th infusion**	**June 21, 2023**	**September 26, 2023** **6th infusion**	**March 23, 2024** **7th infusion** **LFTs of April 24, 2024**
**TB mg/dl**	0.3	-	0.6	1.2	0.77	0.77	0.77	0.9	1.0	0.99	0.75
**DB mg/dl**	0.3	-	0.2	-	0.32	0.19	0.19	-	0.2	0.18	0.14
**IB mg/dl**	0.5	-	0.4	-	0.45	0.58	0.58	-	0.8	0.81	0.61
**AST mg/dl**	20.9	-	40.6	18.1	238	168	161	23	25	28	21
**ALT mg/dl**	37.2	-	91.4	40.8	376	277	240	19	22	25	14
**GGT mg/dl**	37	-	51	-	184	185	188	-	19	20	17
**FA mg/dl**	58	67	78	-	220	192	212	58	61	62	58

## Case report

A 25-year-old male is presented, with a history of RRMS being managed with rituximab. The first and second induction doses were given in June and July 2021, respectively, with no alterations in liver function tests (LFTs) reported during this time. Subsequent semiannual infusions were administered as follows: the third dose on January 12, 2022, and the fourth dose on July 17, 2022. The administration of the fifth dose, scheduled for January 2023, was postponed due to observed alterations in LFTs, as detailed below: BT 0.77, BD 0.32, BI 0.45, AST 238.60, ALT 376, GGT 184, FA 220, PT 7.4, and a referral was made to the hepatology service. During the initial assessment, a pattern indicative of hepatocellular damage was noted, characterized by an R factor of 5.1, accompanied by an elevation of ALT 10 times the upper normal limit. A viral profile was conducted, in which infection by hepatotropic viruses (Hepatitis A, B, C, E), non-hepatotropic viruses (Cytomegalovirus, Ebstein-Barr), hemochromatosis, metabolic, and autoimmune hepatic diseases were ruled out. A liver ultrasound reported normal, only mild signs of fatty liver. It is important to note that the patient did not take any other medications, including supplements and herbal remedies. Due to suspicion of DILI related to rituximab DILI-RECAM 10 points [6], a liver biopsy was performed. The biopsy results were consistent with rituximab-induced liver damage, and excluded alternative etiologies of liver injury (Fig. 1). Treatment with 250 mg of ursodeoxycholic acid (UDCA) every 8 hours (10 mg/kg/day) was initiated. Following this treatment, the patient showed a favorable response, evidenced by the normalization of LFTs. Subsequently, it was decided to continue UDCA treatment as a preventive measure, aiming to sustain its hepatoprotective effects and mitigate the risk of further episodes of DILI secondary to Rituximab. Since then, he received 3 additional infusions without presenting alterations in LFT (table 1).

## Discussion

It is important to note that the literature documents a few cases of DILI due to rituximab (not associated with HBV reactivation), which show an early onset after the first dose of drug induction. The significance of this case lies in its provision of histological evidence and documentation of DILI attributed to rituximab, independent of the HBV association. Notably, the onset of DILI occurred after the fourth infusion, with sustained alterations in LFTs. Therefore, DILI due to rituximab can occur after any infusion, regardless of dose or duration. Some case reports highlight the unpredictable nature of this condition during rituximab therapy, showing that it can arise during therapy, even beyond the induction phase. These findings emphasize the need for careful monitoring and further investigation into the mechanisms of DILI, reinforcing that hepatotoxicity can occur at any point during treatment [3, 7].

Routine monitoring of LFTs before each infusion is strongly recommended to facilitate early detection and intervention, ensuring prompt treatment to mitigate any adverse effects and maintain appropriate therapy.

## Data Availability

Readers can access supporting data by referring to listed references.

## Financing

No sponsorship of any kind was received to carry out this article.
